# Assessment of Aggregated and Exosome-Associated α-Synuclein in Brain Tissue and Cerebrospinal Fluid Using Specific Immunoassays

**DOI:** 10.3390/diagnostics13132192

**Published:** 2023-06-27

**Authors:** Dimitrios Anagnostou, Garifalia Sfakianaki, Katerina Melachroinou, Miltiadis Soutos, Vassilios Constantinides, Nishant Vaikath, Ioanna Tsantzali, George P. Paraskevas, Omar El Agnaf, Kostas Vekrellis, Evangelia Emmanouilidou

**Affiliations:** 1 Laboratory of Biochemistry, Department of Chemistry, National and Kapodistrian University of Athens, 15784 Athens, Greece; dimitrismbg@gmail.com (D.A.); garyfaliasfakianaki@gmail.com (G.S.); 2 Center for Basic Research, Biomedical Research Foundation Academy of Athens, 11527 Athens, Greece; kmelachr@bioacademy.gr (K.M.); miltsout4698@gmail.com (M.S.); 3 Neurochemistry Unit, 1st Department of Neurology, Eginition Hospital, National and Kapodistrian University of Athens, 11528 Athens, Greece; vconstan@med.uoa.gr; 4 Neurological Disorder Research Center, Qatar Biomedical Research Institute (QBRI), Hamad Bin Khalifa University (H.B.K.U.), Qatar Foundation, Doha P.O. Box 34110, Qatar; nvaikath@hbku.edu.qa (N.V.); oelagnaf@hbku.edu.qa (O.E.A.); 5 2nd Department of Neurology, Attikon General University Hospital, School of Medicine, National and Kapodistrian University of Athens, 12462 Athens, Greece; docjo1989@gmail.com (I.T.); geoprskvs44@gmail.com (G.P.P.)

**Keywords:** α-synuclein, exosomes, CSF, Parkinson’s Disease, fibrils, ELISA, synucleinopathies

## Abstract

Even though it is currently well-established that α-synuclein aggregation is closely associated with the pathological events in Parkinson’s disease (PD) and several other neurodegenerative disorders, collectively called synucleinopathies, the mechanistic link between α-synuclein aggregates and the onset and progression of neurodegeneration in these diseases remain unclear. The process of aggregation initiates from a structurally distorted monomer that gradually oligomerizes to generate a repertoire of fibrillar and oligomeric multimers that deposit within diseased cells in the brain. Total α-synuclein has been proposed as a potential biomarker in PD, but most of the studies do not discriminate between distinct α-synuclein conformers. To correlate protein measurements to disease pathology, we have developed a conformation-specific ELISA method that selectively detects fibrillar and oligomeric forms of α-synuclein without cross-reacting with monomers. We have used this assay to determine the levels of aggregated α-synuclein in human and mouse brain tissue as well as in CSF and CSF-derived exosomes from patients with synucleinopathy and control subjects. Our results verify the ability of the new assay to detect aggregated α-synuclein in complex matrices and support the idea that the levels of these conformers are related to the age of onset in PD patients, while CSF analysis showed that these species exist in low abundance in CSF and CSF-derived exosomes. Future studies will be required to fully assess the diagnostic usefulness of this ELISA in synucleinopathies.

## 1. Introduction

A major pathological feature of Parkinson’s disease (PD) is the accumulation and toxic gain of function of misfolded assemblies of α-synuclein gradually generating fibrillar structures that deposit in the PD brain as intraneuronal inclusions called Lewy bodies (LBs). Fibrillar α-synuclein has also been shown to accumulate in dementia with LBs (DLB), and in glial cytoplasmic inclusions in multiple system atrophy (MSA), diseases that are collectively termed synucleinopathies [1]. α-synuclein can exist in various forms, from an unfolded monomer that can bind to a cellular membrane or form tetrameric multimers, to β-sheet oriented fibrils that are thought to be the end point of a stepwise aggregation process. Unlike the compact nature of fibrils, oligomers are soluble and have the capacity to rapidly change conformation, forming a heterogeneous population of conformers both in terms of size and structure [2]. There is compelling evidence to suggest that aggregated forms of α-synuclein, especially newly formed oligomers, are toxic to cells. It has also been shown that oligomeric assemblies of α-synuclein exhibit prion-like properties and spread along interconnected neuronal networks [3]. There is an urgent demand for biomarkers to diagnose such disorders, especially in their early stages when diagnosis is most problematic. The link between α-synuclein and the development of pathology in synucleinopathies, along with the identification of various α-synuclein species in cerebrospinal fluid (CSF), suggest that α-synuclein levels in CSF might be a promising biomarker that could differentially diagnose synucleinopathies [4]. 

A number of studies have investigated the levels of total α-synuclein in CSF; however, they have reported inconsistent findings [5,6,7,8]. It is important to note that the use of total α-synuclein levels as a biomarker for synucleinopathies diagnosis does not take into account the conformation state of α-synuclein, and may thus lack specificity for differential diagnosis. It is plausible that targeting such conformers might be of better diagnostic and therapeutic potential. To this end, various studies have shown that oligomeric α-synuclein levels and the ratio of oligomeric to total α-synuclein are increased in PD patients compared to controls [8,9,10,11]. Still, it should be mentioned that we do not have a clear understanding of the exact nature of native oligomeric species, and we lack specific antibodies to detect such species and differentiate them from other aggregated forms. One other aspect that needs to be considered in this biomarker endeavor is the post-translational modified (PTM) species of α-synuclein, considering that several α-synuclein PTMs have been identified in the postmortem brain tissues of patients with PD and other synucleinopathies [12]. In this respect, phosphorylated α-synuclein at S129 has been shown to be the major species in LBs [13].

α-synuclein has also been detected in CSF-derived extracellular vesicles, termed exosomes, from both PD and DLB patients [14,15,16,17]. Τhe notion that CSF exosomes contain specific cargo, including amyloidogenic proteins indicative of brain pathophysiology, suggests that such vesicles may be potential biomarkers for PD [18]. Given that oligomeric species of α-synuclein have been detected in exosomes [16] and that PD-derived exosomes could aid the oligomerization of α-synuclein [16], one can argue that exosomal α-synuclein species might serve as biomarkers in PD. To this end, lower levels of CSF exosome-associated α-synuclein were observed in PD patients [16], which is consistent with the low total α-synuclein levels reported. Shi et al. [19] demonstrated that plasma levels of exosome associated-α-synuclein were higher in PD patients and correlated with disease severity. In addition, it was recently reported that salivary exosomes isolated from PD patients exhibited higher levels of α-synuclein oligomers; furthermore, the α-synuclein oligomers/total α-synuclein ratio was elevated when compared with controls [20]. A recent meta-analysis that included 13 studies related to exosomal α-synuclein suggested that the levels of the protein in neuronal derived exosomes may indeed differentiate PD and healthy controls [21].

In this study, we utilized conformation-specific antibodies for the development of a sandwich Enzyme-Linked Immunoabsorbent Assay (ELISA) that selectively recognizes α-synuclein fibrils and high-order oligomers without cross-reacting with monomers. The new ELISA was applied for the measurement of these species in brain tissue, CSF samples, and CSF-derived exosomes isolated from synucleinopathy patients. Our results verify the ability of the new assay to detect aggregated α-synuclein in complex matrices and support the idea that the levels of these conformers are related to the age of onset in PD patients. Our CSF analysis showed that these species exist in low abundance in CSF and CSF-derived exosomes; therefore, careful assessment and a large number of samples are required to conclude whether the detection of aggregated α-synuclein in CSF could be of diagnostic usefulness for synucleinopathies.

## 2. Materials and Methods

### 2.1. Patients

A total of 22 patients were included for the purposes of this study. The synucleinopathy group (*n* = 14) included six patients with a diagnosis of probable MSA, four patients with a diagnosis of clinically established PD and four patients with a diagnosis of probable DLB, according to established diagnostic criteria [22,23,24]. The neurological control group included patients with a diagnosis of probable progressive supranuclear palsy (PSP) [25]. The two groups did not differ in their demographic, clinical or neuropsychological profiles (Table 1).

### 2.2. Human Brain Post-Mortem Samples

Tissue corresponding to the putamen and the caudate nucleus brain areas from 8 PD patients and 8 non-PD control individuals were obtained from the PD UK Brain Bank. The non-PD individuals had no life-threatening neurological conditions. The demographic and clinical characteristics of the human post-mortem samples are described in Table 2.

### 2.3. Mice

Adult female and male homozygous A53T α-synuclein transgenic C57BI/C3H mice (A53T Tg, line M83-RRID: IMSR_JAX:004479) and wild-type (Wt) age- and sex-matched littermates were used [26]. Animals were housed in the animal facility of the Biomedical Research Foundation of the Academy of Athens in a room with a controlled light–dark cycle (12 h light–12 h dark) with continuous access to food and water. The brain was excised, and cortices were dissected as previously described [27].

### 2.4. CSF Collection

CSF collection was performed as previously described [28]. Approximately 12 mL of CSF was drawn from each subject and these were used for the experiments of this study and routine diagnostic purposes. CSF was immediately centrifuged at 500× *g* for the removal of cells, aliquoted and stored at −80 °C until analysis. Samples with more than 50 red blood cells as measured in the first tube were rejected.

### 2.5. Isolation of Exosomes from CSF

Exosomes were isolated from pre-cleared concentrated CSF. CSF was pre-cleared by sequential centrifugation at 4000× *g* at 4 °C for 10 min, followed by 10,000× *g* at 4 °C for 30 min. Pre-cleared CSF (1.6 mL) was concentrated 8 times using 3 kDa cut-off filters (Amicon Ultra-4, Merck Millipore, Darmstadt, Germany) and used for exosome isolation using the ExoQuick kit (System Biosciences, Palo Alto, CA, USA) according to the manufacturer’s instructions. Briefly, 50 μL of Exosome Precipitation Solution was added to 200 μL concentrated CSF and exosomes were precipitated at 1500× *g*, for 30 min at 4 °C. Residual solution was removed by an additional centrifugation at 1500× *g*, for 10 min at 4 °C. The exosomal pellet was reconstituted in 20 μL sterile PBS. The enrichment of the pellet in exosomes was confirmed by measuring the acetylcholinesterase activity as previously described [29]. 

### 2.6. Generation of α-Synuclein Fibrils and Oligomers

The generation and characterization of recombinant α-synuclein pre-formed fibrils (PFFs) have been described previously [30]. PFFs were prepared at a concentration of 5 mg/mL and used after their sonication according to established protocols [31]. PFFs solutions used as ELISA calibrators were prepared in 10 mM Tris-Cl, pH 7.6, 100 mM NaCl, 0.1% Tween-20 and 1% BSA (TBS-T/BSA buffer) at a concentration of 50 μg/mL and stored at −80 °C. After two cycles of freezing and thawing, PFF aliquots were discarded. The production and characterization of the stable α-synuclein oligomers in the presence of the lipid peroxidation products, 4-oxo-2-nonenal (ONE oligomers) and 4-hydroxy-2-nonenal (HNE oligomers), have been recently established [32].

### 2.7. ELISA for the Measurement of Aggregated α-Synuclein

For the measurement of α-synuclein aggregates, 96-well plates (Corning Costar, Glendale, AZ, USA) were coated overnight at 25 °C with 1 μg/mL of the mouse monoclonal antibody Syn-F2 (50 μL/well) in 100 mM NaHCO_3_ (pH 9.6). The plates were washed three times with wash buffer (50 mM Tris pH 7.4, 150 mM NaCl, 0.1% Tween-20). Standards and samples were diluted in 10 mM Tris-Cl, pH 7.6, 100 mM NaCl, 0.1% Tween-20 and 1% BSA (TBS-T/BSA buffer) and incubated for 2.5 h at 37 °C. After washing three times, the rabbit monoclonal antibody MJFR-14-6-4-2 (Abcam, Cambridge, UK) was added at a concentration of 74 ng/mL and incubated for 1 h at 25 °C. The wells were washed again three times, and the anti-rabbit IgG-HRP antibody (Agilent, Santa Clara, CA, USA) was applied at a concentration of 16.7 ng/mL (1:15,000 dilution in TBS-T/BSA buffer) for 30 min at 4 °C. The bound HRP was detected by adding 50 μL/well of HRP substrate (Luminata Crescendo ELISA HRP chemiluminescent substrate, Merck Millipore), and the chemiluminescence in relative light units was measured after 5 min in a Biotek Synergy H1 multimode reader (Agilent, Santa Clara, CA, USA).

### 2.8. ELISA for the Measurement of Total α-Synuclein

The development of the ELISA method used to measure total α-synuclein has been described in a previous study [28]. The assay has been previously validated both in cell culture systems [27] and in human biological samples (CSF, plasma, serum) [33,34]. The only modification applied here was the replacement of rabbit polyclonal C-20 detection antibody (Santa Cruz Biotechnology, Dallas, TX, USA), which was discontinued from production, with the rabbit polyclonal PA5-85791 antibody Thermo Fisher Scientific, Waltham, MA, USA) which again targets the C-terminus of α-synuclein. The C-terminal epitope binding is extremely important to provide the steric hindrance required for the simultaneous binding of the detection and Syn1 capture antibodies to the target protein, considering that Syn1 recognizes the 91–99 aa of the protein. The PA5-85791 antibody is also rabbit polyclonal and shows high specificity for the detection of all α-synuclein conformers. Comparison of the two sets of antibodies in our ELISA setup indicated that the PA5-85791 performed similarly to C-20, retaining the analytical characteristics of the assay.

### 2.9. Protein Extraction and Immunoblotting

Human brain tissues were homogenized with a Teflon glass homogenizer in 50 mM Tris-HCl (pH 6.8), 1 mM EDTA supplemented with a mixture of protease and phosphatase inhibitors (1 μΜ pepstatin A, 1 μΜ leupeptin and 0.15 μΜ aprotinin, phosphatase inhibitor cocktail) (A32957, Roche, Basel, Switzerland). Protein extraction was accomplished by the addition of 1% (*w*/*v*) 3-[(3-cholamidopropyl) dimethylammonio]-1-propane-sulphonate (CHAPS) and incubation for 15 min on ice. Protein concentration determination was performed according to the Bradford method (Biorad Laboratories, Hercules, CA, USA) using BSA as a standard.

For protein extraction from cell lysates and mouse cortical tissue, samples were homogenized in a buffer containing 50 mM Tris/HCl (pH 7.6), 150 mM NaCl, 2 mM EDTA supplemented with 1% Triton-X100. The cell lysate was incubated for 30 min at 4 °C and centrifuged at 13,000 rpm for 30 min at 4 °C. The Triton-insoluble pellet was further reconstituted in the same buffer supplemented with 1% SDS. Protein concentration determination was performed according to the DC protein assay (Biorad Laboratories, Hercules, CA, USA) using BSA as a standard.

Homogenates were analyzed by denaturing gel electrophoresis (SDS-PAGE) in Tris-glycine buffer followed by protein transfer to nitrocellulose membranes. Exosomes were homogenized by brief sonication before being analyzed by SDS-PAGE. Primary antibodies against α-synuclein (Syn-1, mouse monoclonal, 1:1000; BD Transductions, and C-20, rabbit polyclonal, 1:1000; Santa Cruz Biotechnology), flotillin-1 (C-7, mouse monoclonal, 1:1000; Santa Cruz, sc-133153), TSG101 (EPR7130(B), rabbit monoclonal, 1:1000; Abcam, ab125011) were applied for 16 h at 4 °C and detected following incubation with a peroxidase-conjugated anti-mouse (1:10,000 dilution; ab205719, Abcam) or anti-rabbit (1:10,000 dilution; ab205718, Abcam) secondary antibody for 1 h. Protein signals were detected using electrogenerated chemiluminescence (ECL) reagents according to the manufacturer’s recommendations (Thermo Fisher Scientific Inc., Whaltham, MA, USA). GAPDH and γ-tubulin were used as loading controls for normalization.

### 2.10. Statistics

Data analysis was carried out using the GraphPad Prism 4 software. All measurements were analyzed with descriptive statistics and results were presented as mean ± Standard Error of the Mean (SEM). Variables were normally distributed as tested by the Shapiro–Wilk test (Shapiro–Wilk test, *p* > 0.05) and statistical analysis was performed by a two-tailed Student’s *t* test. The *p*-value threshold was set at <0.05.

## 3. Results

### 3.1. Development of a Novel ELISA for the Quantification of α-Synuclein Fibrillar and Oligomeric Forms

**Figure 1:**
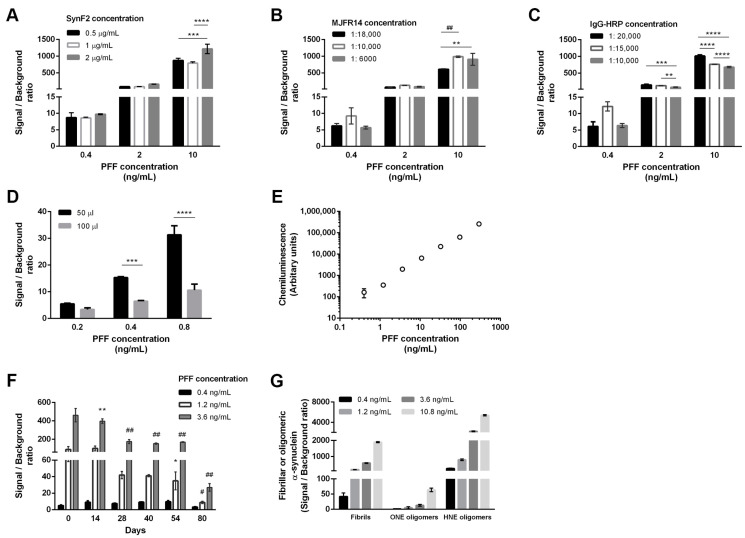
Optimization studies for the development of aggregated α-synuclein ELISA. (**A**) Selection of the optimal concentration for the capture antibody. The ELISA was performed using 0.5, 1.0, 2.0 μg/mL SynF2 antibody and the signal-to-background ratio was assessed in 0.4, 2, 10 ng/mL of PFFs. Statistics were performed comparing the results of the three SynF2 concentrations in each concentration of PFFs using Two-way ANOVA followed by a Tukey’s multiple comparisons test. Significant differences were found only in 10 ng/mL PFFs (*** *p* = 0.0007, **** *p* < 0.0001). (**B**) Selection of the optimal concentration for the detection antibody. The ELISA was performed using 1:18,000, 1:10,000 and 1:6000 dilutions of MJFR14 antibody and the signal-to-background ratio was assessed as previously. Statistics were performed as in (**A**). Significant differences were found only at 10 ng/mL PFFs (** *p* = 0.0075, ^##^ *p* = 0.0010). (**C**) Selection of the optimal concentration for the IgG-HRP antibody. 1:20,000, 1:15,000 and 1:10,000 dilutions of IgG-HRP were tested by assessing the signal-to-background ratio as previously. Statistics were as in (**A**). Significant differences were found in 2 and 10 ng/mL PFFs (** *p* = 0.0041, *** *p* = 0.0001, **** *p* < 0.0001). (**D**) Two sample volumes, 50 and 100 μL, were tested in 0.2, 0.4 and 0.8 ng/mL three concentrations of PFFs. Statistics were performed using Two-way ANOVA followed by a Sidak’s multiple comparisons test (*** *p* = 0.0001, **** *p* < 0.0001). (**E**) Generation of the standard curve for the new aggregated α-synuclein ELISA showing the linear range and the sensitivity of the new assay. Error bars are too low to be depicted and are shown separately in Figure S1. (**F**) Evaluation of the PFFs standard during 80 days using 0.4, 1.2 and 3.6 ng/mL PFFs. Statistics were performed comparing the results at 0 days with the rest time points for a given PFF concentration using Two-way ANOVA followed by Tukey’s multiple comparison test. No significant changes were observed for 0.4 ng/mL PFFs. Statistical significance for 1.2 and 3.6 ng/mL PFFs are denoted (* *p* = 0.0420, ** *p* = 0.0082, ^#^ *p* = 0.0007, ^##^ *p* < 0.0001). (**G**) Assessment of the ability of the new ELISA to measure fibrillar (PFF) and oligomeric (ONE- and HNE-oligomers) forms of α-synuclein.

To develop a conformation-specific immunoassay for aggregated α-synuclein, we used the mouse monoclonal Syn-F2 antibody to capture fibrillar and oligomeric α-synuclein assemblies. The Syn-F2 antibody has been extensively characterized by a variety of biochemical techniques, including dot blot, immunohistochemistry and ELISA, and has been shown to preferentially bind to mature amyloid fibrils and high molecular weight oligomers, either prepared in vitro or found in post-mortem brain sections [30,32]. To ensure that our immunoassay would selectively detect the aggregated α-synuclein species and not monomeric α-synuclein, we used the aggregate-specific rabbit monoclonal antibody MJFR-14-6-4-2 (Abcam) as the detection antibody. The new ELISA was optimized using sonicated PFFs as calibrators. The optimal concentration of Syn-F2 capture antibody was determined by measuring the signal over background ratio for 0.4, 2 and 10 ng/mL PFFs (Figure 1A). Coating the surface of the well with 0.5, 1 or 2 μg/mL Syn-F2 showed similar results for all three PFF concentrations used. In a similar fashion, the concentration of MJFR-14-6-4-2 was optimized and a dilution of 1:10,000 was selected as optimal for all three PFF concentrations tested (Figure 1B). Further, we found that the 1:15,000 dilution of IgG-HRP antibody showed the highest sensitivity for all three PFF concentrations (Figure 1C). Finally, we tested whether changing the sample volume from 50 μL to 100 μL could result in increased sensitivity, but found that the performance of the assay was better when using lower sample volumes (i.e., 50 μL, Figure 1D).

The analytical characteristics of the new ELISA method were determined using the optimized protocol. As demonstrated by the standard curve (Figure 1E and Figure S1), the limit of quantitation was estimated by measuring serial dilutions of PFFs in the range of 0.4–292 ng/mL, providing a detection limit of 0.1 ng/mL with a signal-to-background ratio of 2. The linear range of the assay extends up to 300 ng/mL. The intra-assay coefficient of variance (CV) was <5% (average). The inter-assay CV was estimated by measuring three different PFF concentrations (0.4, 1.2, and 3.6 ng/mL) every week over a period of 80 days (Figure 1F). We observed a significant reduction in the signal-to-background ratio even after 2 cycles of freezing and thawing the PFF solution when measured four weeks after preparation. These results are in agreement with previous observations, suggesting the removal of the MJFR-14-6-4-2 epitope from oligomerized α-synuclein upon repetitive freeze and thaw cycles [35,36]. A day-to-day reproducibility of 8.8% was estimated from five independent measurements of 0.4 ng/mL PFF standard during a period of 54 days. To calculate the intra-assay CV, we compared triplicate measurements from two different PFF concentrations (1.2 and 3.6 ng/mL) in six different plates. The average intra-assay %CV was 4.2% (range 1.1–8.4%). Finally, the ability of the new ELISA to detect oligomeric α-synuclein was assessed using stable ONE- and HNE-oligomers in the range of 0.4–10.8 ng/mL (Figure 1G). Our results indicated that the ELISA could readily detect both oligomeric assemblies. In fact, measurement of HNE-oligomers resulted in high signal-to-background ratios, confirming previous studies [32].

### 3.2. Validation of Fibrillar α-Synuclein ELISA

**Figure 2:**
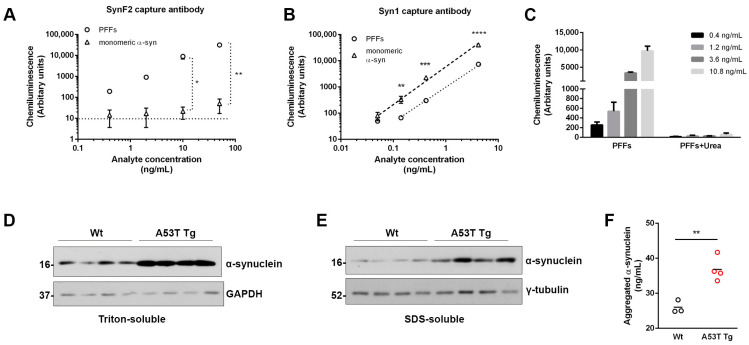
Validation of the ELISA for aggregated α-synuclein. (**A**) The selectivity of the ELISA for the fibrillar forms of α-synuclein was tested by measuring similar concentrations of monomeric α-synuclein. Monomeric α-synuclein was undetectable at concentrations 0.4 and 2.0 ng/mL and barely detectable (signals above background) at 10 and 50 ng/mL. Statistics for 10 and 50 ng/mL were performed by multiple *t*-tests (* *p* = 0.0023 and ** *p* = 0.00036, respectively). (**B**) Monomeric α-synuclein was efficiently measured when SynF2 antibody was replaced with Syn1, which recognizes all forms of α-synuclein. Syn1 also recognized PFFs, although with lower sensitivity. Statistics, as in (**A**), ** *p* = 0.00952, *** *p* = 0.00039, **** *p* < 0.0001. (**C**) The conformation-dependent specificity was assessed by measuring PFFs following urea treatment. Urea-treated samples showed non-detectable chemiluminescence signals. (**D**,**E**) Cortex tissue from Wt and A53T transgenic mice (*N* = 4 per genotype) was differentially fractionated using Triton-X100 and SDS. The Triton-soluble (25 μg per sample) and the SDS-soluble (15 μg per sample) fractions were analyzed by Western blotting using Syn1 antibody. GAPDH and γ-tubulin were used as loading controls. (**F**) Aggregated α-synuclein was measured in the SDS-soluble fractions from Wt and A53T cortical tissue homogenates (*N* = 3 for Wt and *N* = 4 for A53T mice, depicted with black and red circles, respectively) using the new ELISA. Statistics by Student’s *t*-test, ** *p* < 0.01.

Several tests were performed to validate the specificity of the new ELISA. First, we wanted to verify that the selected antibodies bind to aggregated α-synuclein conformers without detecting the monomeric protein. For this, we used the ELISA to measure 0.4–50 ng/mL PFFs or α-synuclein monomer (Figure 2A). The signal obtained for monomeric α-synuclein did not exceed the background signal in all concentrations tested except the highest concentration (50 ng/mL), which only exhibited a low signal, indicating that the configuration used was specific for the detection of fibrillar and oligomeric α-synuclein. As a control, the commercially available mouse monoclonal antibody Syn1, which is known to bind all α-synuclein conformers (fibrils, oligomers, monomers), was found to detect both PFFs and monomeric α-synuclein (Figure 2B). To further confirm that the ELISA is aggregation and conformational specific, we denatured PFFs using 8 M urea for 3 h at room temperature. PFFs and urea-denatured PFFs were subsequently analyzed by the ELISA (Figure 2C). Our results showed that pre-treatment with urea completely abolished the signal obtained.

The new ELISA was also validated using brain tissue material from transgenic mice and human post-mortem samples. We dissected the cortices from four adult A53T α-synuclein transgenic mice (M83 line, A53T Tg mice) and four age-matched wild type littermates (Wt mice). The A53T Tg mice express the human A53T mutated α-synuclein under the control of the prion promoter and have been extensively characterized as a mouse model for PD. The expression of the A53T transgene leads to the generation and build-up of distinct oligomeric α-synuclein species of variable size and solubility. Tissue proteins were serially detergent-extracted using first 1% Triton-X100 to collect the soluble proteins, and then 1% SDS to obtain the more insoluble proteins. By using this protocol, soluble α-synuclein is obtained in the Triton-X soluble fraction (Figure 2D and Figure S2), whereas aggregated α-synuclein is recovered in the SDS soluble fraction (Figure 2E and Figure S2). We measured α-synuclein in the Triton-soluble fractions with our in-house ELISA. The samples were prepared in a concentration of 3 mg/mL and assessed in a concentration of 0.15 mg/mL TBS-T/BSA buffer with the ELISA. The results indeed verified the increased levels of aggregated α-synuclein in the A53T Tg mice compared with Wt mice (Figure 2F).

### 3.3. Assessment of Aggregated α-Synuclein in Human Post-Mortem Brain Tissue

**Figure 3:**
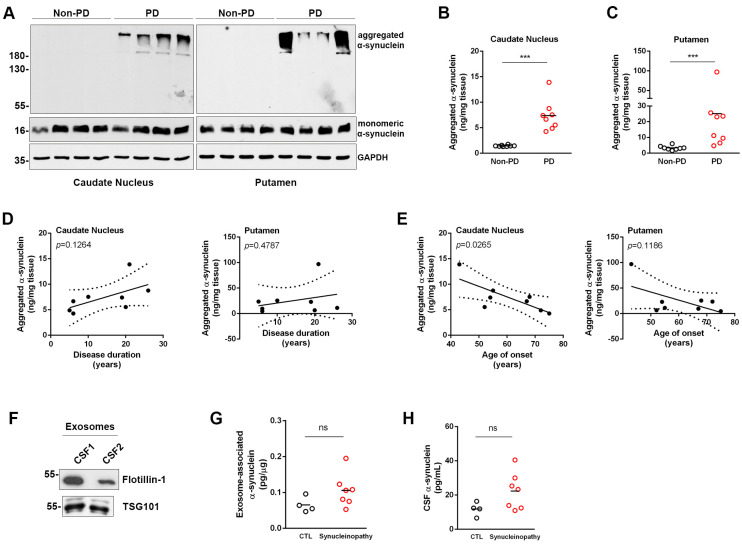
Assessment of aggregated α-synuclein in human post-mortem tissue, CSF and CSF-derived exosomes. (**A**) Tissue homogenates (30 μg per sample) corresponding to the caudate nucleus and putamen from PD patients and non-PD control individuals were analyzed for the detection of α-synuclein species by Western blotting using C-20 antibody (*N* = 8 per group per brain area). GAPDH was used as a loading control. (**B**,**C**) The same homogenates were analyzed by the new ELISA. Statistics by Student’s *t*-test; *** *p* < 0.001. (**D**) Correlation analysis of the levels of aggregated α-synuclein with disease duration in the caudate nucleus (*R*^2^ = 0.3500) and putamen (*R*^2^ = 0.08679). (**E**) Correlation analysis of the levels of aggregated α-synuclein with the age of disease onset in the caudate nucleus (*R*^2^ = 0.5874) and putamen (*R*^2^ = 0.3557). (**F**) Extracellular vesicles isolated from CSF were analyzed by Western blotting for the presence of the exosome markers, flotillin-1 and TSG101. A representative blot showing vesicles from two CSF samples is shown. (**G**,**H**) Total α-synuclein was assessed in CSF exosomes and exosome-depleted CSF samples from control subjects (*N* = 4, black circles) and patients with synucleinopathies (*N* = 7, red circles). Statistics by Student’s *t*-test; ns: non-significant differences, solid lines indicates mean values.

To further validate the ability of the new ELISA method to detect disease-related α-synuclein aggregates, we analyzed post-mortem brain tissue corresponding to the caudate nucleus and putamen from eight PD patients and eight non-PD individuals. In this case, proteins were extracted using the detergent CHAPS to retain the conformational characteristics of the proteins. Immunoblotting analysis of these samples revealed the presence of insoluble high molecular weight aggregated α-synuclein only in the PD samples, whereas the levels of monomeric α-synuclein remained unaltered between PD and non-PD samples (statistical comparison between densitometric values of monomeric α-synuclein in non-PD and PD samples was performed by Student’s *t*-test, *p* = 0.8613 and *p* = 0.4783, for caudate nucleus and putamen, respectively) (Figure 3A). For the assessment of aggregated α-synuclein in the caudate nucleus homogenates, 12.5 μg and 3 μg were used for the non-PD and PD groups, respectively. For the putamen samples, 10 μg of tissue per well were assessed. Measurement of human brain homogenates with our ELISA accurately reflected the significant increase in the levels of aggregated α-synuclein in the PD vs. non-PD subjects in both brain areas tested (Figure 3B,C). Our data suggested that even though aggregated α-synuclein was present in both brain regions analyzed, its levels were mostly elevated in the putamen (7.38 ± 3.03 vs. 25.19 ± 30.21 ng aggregated α-synuclein/mg tissue in the caudate nucleus and putamen of PD patients, respectively). We only measured minimal levels of aggregated α-synuclein in the brain homogenates from non-PD individuals (1.46 ± 0.15 and 3.37 ± 1.34 ng/mg tissue in the caudate nucleus and putamen of PD patients, respectively). Subsequent analysis did not show any correlation between the levels of aggregated α-synuclein and disease duration (Figure 3D), but revealed a significant negative correlation with the age of onset in the caudate nucleus (Figure 3E).

### 3.4. Assessment of Aggregated and Total α-Synuclein in CSF and CSF-Derived Exosomes

Aggregated α-synuclein has been linked with the development of pathology in synucleinopathies and has been explored as a potential biomarker for diagnosis and progression status in these diseases. Although recent evidence supports that CSF total α-synuclein is significantly increased in patients with tauopathies compared with patients with synucleinopathies, the CSF load of oligomeric α-synuclein in these patient groups showed no differences. To understand whether the detection of aggregated α-synuclein in CSF could have any clinical relevance, we used the aggregate-specific ELISA to assess the levels of fibrillar and oligomeric α-synuclein in a small number of CSF samples from patients with typical AD (*N* = 7), synucleinopathies such as PD, MSA, and DLB (*N* = 10) and non-neurological control subjects (*N* = 7). The results are summarized in Table 3. For the measurement in CSF samples, 45 μL of CSF per well were used, supplemented with 5 μL of 10× TBS-T/BSA buffer. Only 8 of the 24 CSF samples showed detectable signals corresponding to aggregated α-synuclein, suggesting that these species are present in extremely low levels. To exclude the possibility that the lack of signal in the majority of the CSF samples was due to unsatisfactory assay sensitivity, we used a 3 kDa cut-off filter to concentrate one CSF sample with undetectable levels of aggregated α-synuclein. Assessment of the 4-times-concentrated CSF with the ELISA did not result in any signal, further indicating the low abundance of aggregated α-synuclein in the CSF.

We next wanted to investigate whether CSF exosomes, extracellular vesicles of endocytic origin, could carry aggregated α-synuclein. Intercellular communication through exosomes is considered to be involved in the pathogenesis of various disorders, including neurodegenerative diseases. High-order oligomeric conformers of α-synuclein are found to localize in exosomes in cell culture systems [37], and brain-derived exosomal α-synuclein has been proposed as a potential biomarker for PD [38]. To assess neuronal exosome-associated α-synuclein, we isolated extracellular vesicles from CSF samples from patients with synucleinopathies (PD, DLB and MSA, *N* = 14) and control individuals (*N* = 5) using an ExoQuick kit, homogenized vesicles by sonication and measured aggregated and total α-synuclein using our ELISA. CSF vesicles were enriched in exosomes, as indicated by the presence of two exosome-specific markers, flotillin-1 and TSG101 (Figure 3F). To measure aggregated α-synuclein with the ELISA, 5 μg of extracellular vesicles were used. However, we could not detect aggregated α-synuclein in any of the exosome preparations consistent with the low abundance of fibrillar and oligomeric species in the CSF. We used all the exosomes isolated from 1.6 mL CSF (12–50 μg) and measured total α-synuclein in 11 out of the 19 samples we tested, and did not find significant differences between control subjects and synucleinopathy patients (Table 3 and Figure 3G). The levels of total α-synuclein in the corresponding exosome-depleted CSF samples also followed a similar trend (11.85 ± 2.01 and 22.36 ± 4.07 pg/mL for control and synucleinopathy samples, respectively) (Figure 3H).

## 4. Discussion

α-synuclein aggregation is closely linked with the pathological events in PD, PD with dementia (PDD), MSA and DLB, collectively called synucleinopathies. Despite the undeniable mechanistic connection between α-synuclein aggregation and neurodegeneration in PD, the nature of the aberrant conformers that mediate neurotoxicity in the human brain is still unclear. Current diagnosis of synucleinopathies relies mostly on the appreciation of the clinical symptoms in conjunction with neuroimaging, which could be beneficial for some, but not all, occasions. Although much effort has been invested in the discovery of CSF biomarkers for the improvement of disease diagnostic accuracy, no reliable prognostic and/or diagnostic biomarkers are currently available to allow early and differential diagnosis at time points when pathology is relatively confined and possibly more approachable to therapeutic interventions [4]. α-synuclein has been proposed as a promising biomarker in PD, since α-synuclein aggregation is thought to reflect the pathobiology of the disease in the CNS. In this respect, we have developed a new ELISA for the selective detection of α-synuclein fibrils and oligomers. The chemiluminescent detection of the assay is based on two conformation-specific well-characterized α-synuclein antibodies, Syn-F2 and MJFR-14-6-4-2, that bind to fibrils and high-order oligomers, respectively. Even though these conformation-specific antibodies have been previously established, this is the first time that they have been used in combination, in a sandwich ELISA format, which is why we have used the term “new” to describe this assay. We have validated the new method using brain tissue from M83 transgenic mice, which over-express the A53T variant of the human α-synuclein, and human post-mortem tissue from disease-affected brain areas from PD patients. Finally, we analyzed CSF and CSF-derived exosomes from patients with AD or synucleinopathies and control individuals to investigate whether we could detect aggregated α-synuclein in these samples using the new ELISA.

Our results indicated that the new ELISA is selective for the detection of aggregated α-synuclein, either in the fibrillar or the oligomeric state, and does not detect monomeric α-synuclein. Denaturation of the fibrils used as calibrators resulted in complete loss of the chemiluminescent signal, suggesting that the detection depends on α-synuclein conformational properties. Using sonicated PFFs as calibrators, the estimated sensitivity of the assay was 0.1 ng/mL, which is comparable to the oligomeric ELISAs developed in other studies. The low intra-and inter-assay coefficients of variability (% CV < 5) indicated high accuracy and consistency in the measurements between sample replicates, either in the same plate or in different plates.

α-synuclein is an abundant protein in the normal brain; however, certain conditions can locally promote its oligomerization and aggregation. The aggregates, which gradually generate inclusion bodies, can compromise cellular homeostasis systems and propagate pathology by a series of release–uptake events in synaptically connected brain areas. It is well established that these high molecular weight conformers, but not the low molecular or monomeric proteins, define disease pathology in PD and other synucleinopathies. In support of this, previous studies in human post-mortem tissue report a significant increase in insoluble α-synuclein fraction in PD patients compared to controls, whereas soluble α-synuclein remains unaltered [39]. Our Western blotting analysis also confirmed this idea, showing the presence of high-molecular SDS-insoluble conformers only in the brain tissue from PD patients, whereas the levels of monomeric α-synuclein were similar between non-PD and PD samples. Using the developed ELISA, we measured a significant increase in the levels of aggregated α-synuclein in the caudate nucleus and putamen from PD patients compared with non-PD subjects. This increase was more profound in the putamen. Brain tissue samples from PD patients showed high variability (range: 4.8–97.1 ng/mg tissue for the putamen), suggesting that the accumulation of aggregated α-synuclein probably depends on multiple parameters, including age of onset and disease duration. In fact, we found that the age of onset was negatively correlated with the levels of the aggregated α-synuclein in the caudate nucleus. A similar trend was identified in the putamen, although it did not reach statistical significance. Importantly, we discovered that brain tissue from control individuals also contained fibrillar and oligomeric α-synuclein, albeit at very low levels. Such basal levels in the control tissue were consistent and independent from the age of the individuals.

We have also measured aggregated α-synuclein in CSF samples from patients with AD and synucleinopathies, as well as from neurological control individuals. With our method, aggregated α-synuclein could be detected only in 8 of the 24 samples analyzed. Even though this indicates the low abundance of aggregated α-synuclein in the CSF, the low number of samples limits safe comparisons between the groups. Our results are complementary to the recent study of Constantinides et al. [7], in which CSF oligomeric α-synuclein was measured at low pg levels and showed no differences between patients with synucleinopathies and tauopathies. In general, the assessment of CSF oligomeric α-synuclein levels has been addressed by a limited number of studies, most of them showing higher concentrations of oligomeric α-synuclein in PD compared with controls. However, the diagnostic accuracy of oligomeric α-synuclein remains unsatisfactory for clinical use, even when the ratio of oligomeric-to-total α-synuclein is considered. Importantly, only a few studies provided a blood contamination cut-off which could account for the inconsistency observed among the different studies. To overcome low abundancy and assay sensitivity issues, Majbour et al. recently combined seed amplification assay (SAA) and oligomers-specific ELISA, providing a multiplex test that could reflect disease severity in patients with PD [40]. SAAs, such as Protein-Misfolding Cyclic Amplification (PMCA), Real-Time Quaking-Induced Conversion (RT-QuIC), and HANdai Amyloid Burst Inducer, measure the amount of misfolded proteins that exhibit a prion-like behavior in solution and have been used to assess α-synuclein aggregates in CSF from patients with PD, MSA, DLB and AD, with remarkable diagnostic accuracy [40,41,42,43]. The potential utility of these techniques in a clinical setting for early and differential diagnosis of synucleinopathies is still under investigation.

Further, we have assessed the presence of fibrillar and oligomeric α-synuclein in exosomes isolated from CSF from patients with synucleinopathies and control subjects. In a recent study, CSF-derived extracellular vesicles from PD patients that carried increased levels of α-synuclein were intranasally administered in healthy mice and induced α-synuclein aggregation and PD-like symptoms in vivo, thereby suggesting that these vesicles can mediate the transfer of a neurotoxic cargo [42]. In our study, no aggregated α-synuclein could be detected in CSF exosomes, even though we cannot rule out the possibility that their levels could have been too low to be captured by our assay in the number of vesicles tested (5 μg). Assessment of a higher number of exosomes is required to allow safe conclusions about the presence or absence of aggregated α-synuclein species in these vesicles. The measurement of aggregated α-synuclein in CSF exosomes is also limited by the low number of samples assessed, including only 5 controls and 14 synucleinopathy cases. Accordingly, exosome-associated total α-synuclein was quantified in the majority of CSF samples when a higher number of exosomes (30 μg) was used. The concentration of total α-synuclein was higher in exosomes from patients with synucleinopathy compared with control subjects, although this increase did not reach statistical significance. Importantly, the total α-synuclein levels measured in the corresponding CSF samples showed a similar trend for increase, suggesting that exosomes reflect the molecular composition of CSF, at least regarding α-synuclein content.

In recent years, reliable methodologies that assess the pathology-linked aggregated α-synuclein species have emerged. These include the direct detection of aggregates using conformation-specific antibodies in sandwich ELISA configurations and seed amplification assays that measure the amount of species exhibiting a high potential to act as seeds, thereby templating aggregation. Aggregate-specific ELISAs, when applied to dilute clinical samples, are limited by the low concentration of these conformers relative to the monomeric (or low molecular weight) protein. Our work verifies this concept, since the ELISA proposed here could not detect aggregated α-synuclein in CSF samples due to their low abundance. Assessment of concentrated CSF samples could demonstrate the true concentration of these aggregates and allow the optimization of our method, so as to achieve their quantification. Another limitation of our study is the low number of clinical samples used; especially in the case of CSF-derived exosomes, we found that a larger starting volume per CSF sample is required to isolate EVs with the efficiency and reproducibility required for the clinical application of this protocol.

In conclusion, we have developed an ELISA method of high sensitivity and reproducibility for the selective detection of aggregated α-synuclein in human and mouse brain tissue as well as in CSF and CSF-derived exosomes. An advantage of the assay is that it targets both fibrillar and oligomeric forms of α-synuclein (herein termed as aggregated α-synuclein), providing a means to assess both α-synuclein species considered to possess neurotoxic activity in the context of human disease. Although our study is limited by the low number of clinical samples, our aim was to perform the measurement of aggregated α-synuclein in the CSF with minimum interference in the sample, thus reflecting the protein content as accurately as possible.

## Figures and Tables

**Table 1:** Demographic, clinical and neuropsychological profiles of the synucleinopathy group and the neurological control group of the Eginition Hospital cohort. All data are presented as median (25th–75th quartile). MMSE: Mini Mental State Examination; FAB: Frontal Assessment Battery; UPDRS: Unified Parkinson’s Disease Rating Scale; *: x^2^ test; ‡: Mann–Whitney U test.

	Synucleinopathy	Neurological Controls	*p*-Value
Gender (m/f)	6/8	6/2	0.145 *
Age (years)	64.5 (57–68)	68.5 (61–69)	0.368 ‡
Disease duration (months)	36 (30–60)	30 (24–36)	0.220 ‡
MMSE	28 (24–29)	25 (23–29)	0.607 ‡
FAB	14 (12–15)	11.5 (8–12)	0.066 ‡
UPDRS III	21 (0–51)	16.5 (10–26)	1.000 ‡

**Table 2:** Characteristics of human brain samples.

	Non PD	PD
Age of death ± STDEV	83 ± 9	75 ± 5
Sex (male) %	50	87.5
Disease duration (years) ± STDEV	-	14 ± 8 (range 5–26)
Age of onset (years) ± STDEV	-	61 ± 11 (range 43–75)

**Table 3:** Levels of aggregated and exosomal α-synuclein in CSF samples.

	Non Neurological CTLs	AD	Synucleinopathy
Aggregated α-synuclein (ng/mL)	0.268 ± 0.013 (*N* = 2/7)	0.414 ± 0.114 (*N* = 4/7)	0.644 ± 0.183 (*N* = 2/10)
α-synuclein associated with CSF exosomes (pg/μg)	0.065 ± 0.011 (*N* = 4/5)	-	0.106 ± 0.017 (*N* = 7/14)

## Data Availability

The data presented in the study are available upon request from the corresponding author.

## Supplementary Materials

The following supporting information can be downloaded at: https://www.mdpi.com/article/10.3390/diagnostics13132192/s1, Figure S1: Standard deviation of points in the calibration curve of the assay. Figure S2: Triton- and SDS-soluble α-synuclein in A53T transgenic mice (full blots).

## References

[1] Goedert M., Jakes R., Spillantini M.G. (2017). The Synucleinopathies: Twenty Years On. J. Parkinsons. Dis..

[2] Du X., Xie X., Liu R. (2020). The Role of α -Synuclein Oligomers in Parkinson’s Disease. Int. J. Mol. Sci..

[3] Braak H., Del Tredici K., Rüb U., De Vos R.A.I., Jansen Steur E.N.H., Braak E. (2003). Staging of Brain Pathology Related to Sporadic Parkinson’s Disease. Neurobiol. Aging.

[4] Parnetti L., Gaetani L., Eusebi P., Paciotti S., Hansson O., El-Agnaf O., Mollenhauer B., Blennow K., Calabresi P. (2019). CSF and Blood Biomarkers for Parkinson’s Disease. Lancet Neurol..

[5] Delgado-Alvarado M., Dacosta-Aguayo R., Navalpotro-Gómez I., Gago B., Gorostidi A., Jiménez-Urbieta H., Quiroga-Varela A., Ruiz-Martínez J., Bergareche A., Rodríguez-Oroz M.C. (2018). Ratios of Proteins in Cerebrospinal Fluid in Parkinson’s Disease Cognitive Decline: Prospective Study. Mov. Disord..

[6] Lim X., Yeo J.M., Green A., Pal S. (2013). The Diagnostic Utility of Cerebrospinal Fluid Alpha-Synuclein Analysis in Dementia with Lewy Bodies—A Systematic Review and Meta-Analysis. Park. Relat. Disord..

[7] Constantinides V.C., Majbour N.K., Paraskevas G.P., Abdi I., Safieh-Garabedian B., Stefanis L., El-Agnaf O.M., Kapaki E. (2021). Cerebrospinal Fluid α-Synuclein Species in Cognitive and Movements Disorders. Brain Sci..

[8] Tokuda T., Qureshi M.M., Ardah M.T., Varghese S., Shehab S.A.S., Kasai T., Ishigami N., Tamaoka A., Nakagawa M., El-Agnaf O.M.A. (2010). Detection of Elevated Levels of α-Synuclein Oligomers in CSF from Patients with Parkinson Disease. Neurology.

[9] Majbour N.K., Vaikath N.N., Van Dijk K.D., Ardah M.T., Varghese S., Vesterager L.B., Montezinho L.P., Poole S., Safieh-Garabedian B., Tokuda T. (2016). Oligomeric and Phosphorylated Alpha-Synuclein as Potential CSF Biomarkers for Parkinson’s Disease. Mol. Neurodegener..

[10] Hansson O., Hall S., Öhrfelt A., Zetterberg H., Blennow K., Minthon L., Nägga K., Londos E., Varghese S., Majbour N.K. (2014). Levels of Cerebrospinal Fluid α-Synuclein Oligomers Are Increased in Parkinson’s Disease with Dementia and Dementia with Lewy Bodies Compared to Alzheimer’s Disease. Alzheimer’s Res. Ther..

[11] Parnetti L., Chiasserini D., Persichetti E., Eusebi P., Varghese S., Qureshi M.M., Dardis A., Deganuto M., De Carlo C., Castrioto A. (2014). Cerebrospinal Fluid Lysosomal Enzymes and Alpha-Synuclein in Parkinson’s Disease. Mov. Disord..

[12] Schweighauser M., Shi Y., Tarutani A., Kametani F., Murzin A.G., Ghetti B., Matsubara T., Tomita T., Ando T., Hasegawa K. (2020). Structures of α-Synuclein Filaments from Multiple System Atrophy. Nature.

[13] Oueslati A. (2016). Implication of Alpha-Synuclein Phosphorylation at S129 in Synucleinopathies: What Have We Learned in the Last Decade?. J. Parkinson’s Dis..

[14] Harischandra D.S., Rokad D., Neal M.L., Ghaisas S., Manne S., Sarkar S., Panicker N., Zenitsky G., Jin H., Lewis M. (2019). Manganese Promotes the Aggregation and Prion-like Cell-to-Cell Exosomal Transmission of α-Synuclein. Sci. Signal..

[15] Guo M., Wang J., Zhao Y., Feng Y., Han S., Dong Q., Cui M., Tieu K. (2020). Microglial Exosomes Facilitate A-Synuclein Transmission in Parkinson’s Disease. Brain.

[16] Stuendl A., Kunadt M., Kruse N., Bartels C., Moebius W., Danzer K.M., Mollenhauer B., Schneider A. (2016). Induction of α-Synuclein Aggregate Formation by CSF Exosomes from Patients with Parkinson’s Disease and Dementia with Lewy Bodies. Brain.

[17] Ngolab J., Trinh I., Rockenstein E., Mante M., Florio J., Trejo M., Masliah D., Adame A., Masliah E., Rissman R.A. (2017). Brain-Derived Exosomes from Dementia with Lewy Bodies Propagate α-Synuclein Pathology. Acta Neuropathol. Commun..

[18] Vella L.J., Hill A.F., Cheng L. (2016). Focus on Extracellular Vesicles: Exosomes and Their Role in Protein Trafficking and Biomarker Potential in Alzheimer’s and Parkinson’s Disease. Int. J. Mol. Sci..

[19] Shi M., Liu C., Cook T.J., Bullock K.M., Zhao Y., Ginghina C., Li Y., Aro P., Dator R., He C. (2014). Plasma Exosomal α-Synuclein Is Likely CNS-Derived and Increased in Parkinson’s Disease. Acta Neuropathol..

[20] Rani K., Mukherjee R., Singh E., Kumar S., Sharma V., Vishwakarma P., Bharti P.S., Nikolajeff F., Dinda A.K., Goyal V. (2019). Neuronal Exosomes in Saliva of Parkinson’s Disease Patients: A Pilot Study. Park. Relat. Disord..

[21] Taha H.B., Ati S.S. (2023). Evaluation of α -Synuclein in CNS-Originating Extracellular Vesicles for Evaluation of α -Synuclein in CNS-Originating Extracellular Vesicles for Parkinsonian Disorders: A Systematic Review and Meta-Analysis. Park. Relat. Disord..

[22] McKeith I.G., Boeve B.F., Dickson D.W., Halliday G., Aarsland D., Attems J., Ballard C.G., Bayston A., Beach T.G., Chen-plotkin A. (2017). Diagnosis and Management of Dementia with Lewy Bodies: Fourth Consensus Report of the DLB Consortium. Neurology.

[23] Postuma R.B., Berg D., Stern M., Poewe W., Olanow C.W., Oertel W., Obeso J., Marek K., Litvan I., Lang A.E. (2015). MDS Clinical Diagnostic Criteria for Parkinson’s Disease. Mov. Disord..

[24] Gilman S., Wenning G.K., Low P.A., Brooks D.J., Mathias C.J., Trojanowski J.Q., Wood N.W., Colosimo C., Dürr A., Fowler C.J. (2008). Second Consensus Statement on the Diagnosis of Multiple System Atrophy. Neurology.

[25] Höglinger G.U., Respondek G., Stamelou M., Kurz C., Josephs K.A., Lang A.E., Mollenhauer B., Müller U., Nilsson C., Whitwell J.L. (2017). Clinical Diagnosis of Progressive Supranuclear Palsy: The Movement Disorder Society Criteria. Mov. Disord..

[26] Giasson B.I., Duda J.E., Quinn S.M., Zhang B., Trojanowski J.Q., Lee V.M.-Y. (2002). Neuronal-Synucleinopathy with Severe Movement Disorder in Mice Expressing A53T Human-Synuclein. Neuron.

[27] Emmanouilidou E., Minakaki G., Keramioti M.V., Xylaki M., Balafas E., Chrysanthou-Piterou M., Kloukina I., Vekrellis K. (2016). GABA Transmission via ATP-Dependent K+ Channels Regulates α-Synuclein Secretion in Mouse Striatum. Brain.

[28] Kapaki E., Paraskevas G.P., Emmanouilidou E., Vekrellis K. (2013). The Diagnostic Value of CSF α-Synuclein in the Differential Diagnosis of Dementia with Lewy Bodies vs. Normal Subjects and Patients with Alzheimer’s Disease. PLoS ONE.

[29] Papadopoulos V.E., Nikolopoulou G., Antoniadou I., Karachaliou A., Arianoglou G., Emmanouilidou E., Sardi S.P., Stefanis L., Vekrellis K. (2018). Modulation of β-Glucocerebrosidase Increases α-Synuclein Secretion and Exosome Release in Mouse Models of Parkinson’s Disease. Hum. Mol. Genet..

[30] Vaikath N.N., Majbour N.K., Paleologou K.E., Ardah M.T., van Dam E., van de Berg W.D.J., Forrest S.L., Parkkinen L., Gai W.P., Hattori N. (2015). Generation and Characterization of Novel Conformation-Specific Monoclonal Antibodies for α-Synuclein Pathology. Neurobiol. Dis..

[31] Karampetsou M., Ardah M.T., Semitekolou M., Polissidis A., Samiotaki M., Kalomoiri M., Majbour N., Xanthou G., El-Agnaf O.M.A., Vekrellis K. (2017). Phosphorylated Exogenous Alpha-Synuclein Fibrils Exacerbate Pathology and Induce Neuronal Dysfunction in Mice. Sci. Rep..

[32] Vaikath N., Sudhakaran I., Abdi I., Gupta V., Majbour N., Ghanem S., Abdesselem H., Vekrellis K., El-Agnaf O. (2022). Structural and Biophysical Characterization of Stable Alpha-Synuclein Oligomers. Int. J. Mol. Sci..

[33] Emmanouilidou E., Papagiannakis N., Kouloulia S., Galaziou A., Antonellou R., Papadimitriou D., Athanasiadou A., Bozi M., Koros C., Maniati M. (2020). Peripheral Alpha-Synuclein Levels in Patients with Genetic and Non-Genetic Forms of Parkinson’s Disease. Park. Relat. Disord..

[34] Bougea A., Stefanis L., Emmanouilidou E., Vekrelis K., Kapaki E. (2020). High Discriminatory Ability of Peripheral and CFSF Biomarkers in Lewy Body Diseases. J. Neural Transm..

[35] Umemoto A., Yagi H., So M., Goto Y. (2014). High-Throughput Analysis of Ultrasonication-Forced Amyloid Fibrillation Reveals the Mechanism Underlying the Large Fluctuation in the Lag Time. J. Biol. Chem..

[36] Berkhoudt Lassen L., Gregersen E., Kathrine Isager A., Betzer C., Hahn Kofoed R., Henning Jensen P. (2018). ELISA Method to Detect α-Synuclein Oligomers in Cell and Animal Models. PLoS ONE.

[37] Emmanouilidou E., Melachroinou K., Roumeliotis T., Garbis S.D., Ntzouni M., Margaritis L.H., Stefanis L., Vekrellis K. (2010). Cell-Produced α-Synuclein Is Secreted in a Calcium-Dependent Manner by Exosomes and Impacts Neuronal Survival. J. Neurosci..

[38] Herman S., Djaldetti R., Mollenhauer B., Offen D. (2023). CSF-Derived Extracellular Vesicles from Patients with Parkinson’s Disease Induce Symptoms and Pathology. Brain.

[39] Wills J., Jones J., Haggerty T., Duka V., Joyce J.N., Sidhu A. (2010). Elevated Tauopathy and Alpha-Synuclein Pathology in Postmortem Parkinson’s Disease Brains with and without Dementia. Exp. Neurol..

[40] Majbour N., Aasly J., Abdi I., Ghanem S., Erskine D., Van De Berg W., El-Agnaf O. (2022). Disease-Associated α-Synuclein Aggregates as Biomarkers of Parkinson Disease Clinical Stage. Neurology.

[41] Fairfoul G., McGuire L.I., Pal S., Ironside J.W., Neumann J., Christie S., Joachim C., Esiri M., Evetts S.G., Rolinski M. (2016). Alpha-Synuclein RT-QuIC in the CSF of Patients with Alpha-Synucleinopathies. Ann. Clin. Transl. Neurol..

[42] Shahnawaz M., Tokuda T., Waraga M., Mendez N., Ishii R., Trenkwalder C., Mollenhauer B., Soto C. (2017). Development of a Biochemical Diagnosis of Parkinson Disease by Detection of α-Synuclein Misfolded Aggregates in Cerebrospinal Fluid. JAMA Neurol..

[43] Groveman B.R., Orrù C.D., Hughson A.G., Raymond L.D., Zanusso G., Ghetti B., Campbell K.J., Safar J., Galasko D., Caughey B. (2018). Rapid and Ultra-Sensitive Quantitation of Disease-Associated α-Synuclein Seeds in Brain and Cerebrospinal Fluid by ASyn RT-QuIC. Acta Neuropathol. Commun..

