# Supplementary figures and images for: Assessment of Aggregated and Exosome-Associated α-Synuclein in Brain Tissue and Cerebrospinal Fluid Using Specific Immunoassays
Source: Diagnostics (Basel). 2023 Jun 27;13(13):2192. doi: 10.3390/diagnostics13132192 (PMC10340737; doi:10.3390/diagnostics13132192)

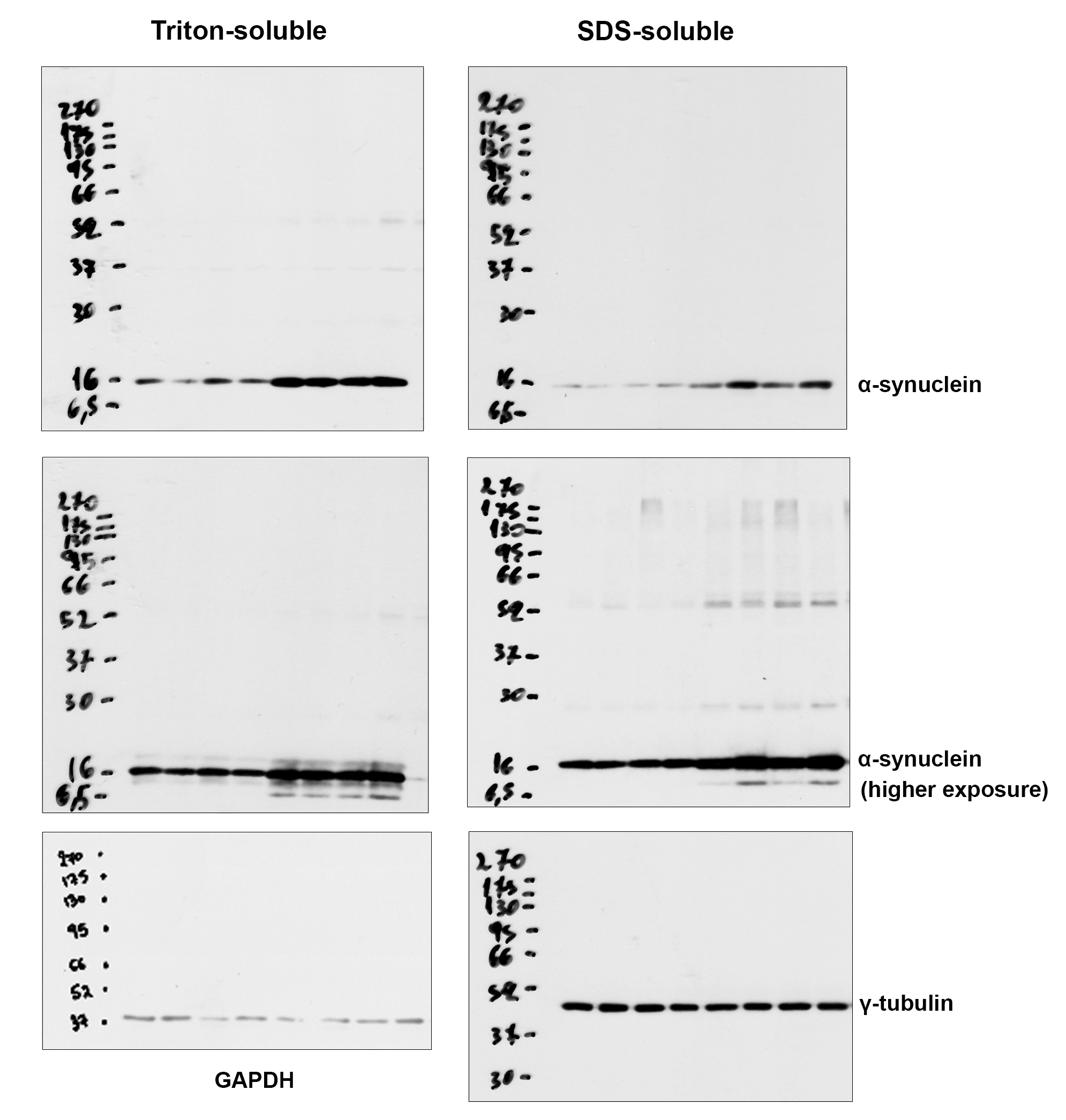

Supplement: Supplementary file 1 [file diagnostics-13-02192-s001.zip › Figure S1. Standard deviation of points in the calibration curve of the assay.tif]

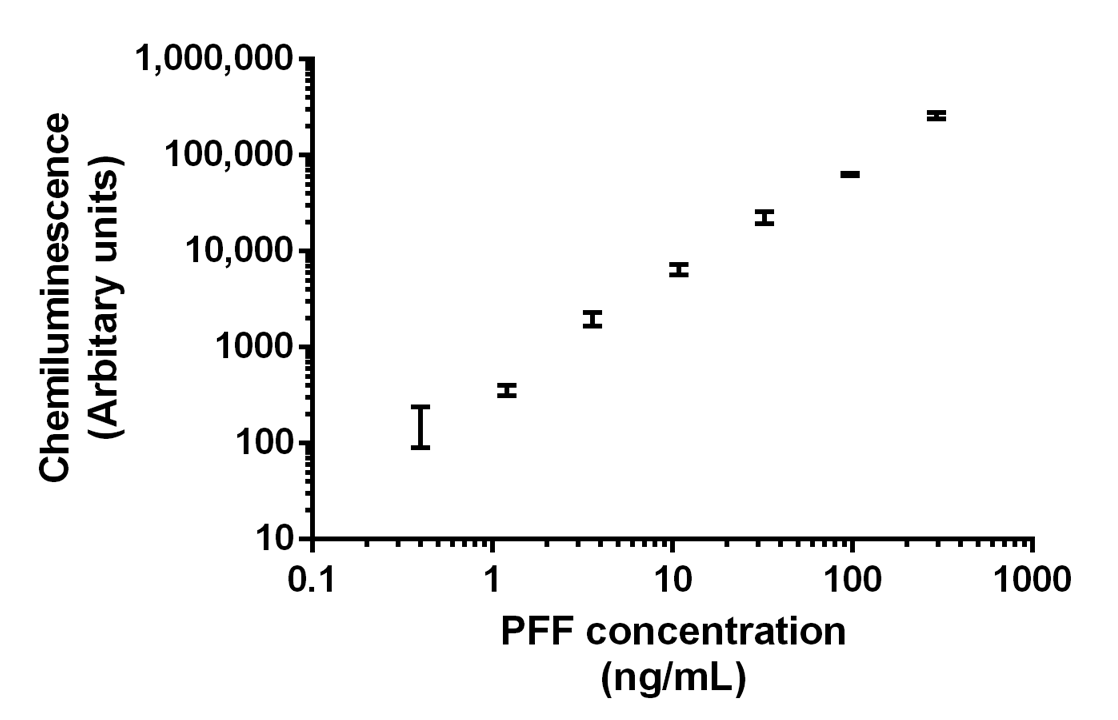

Supplement: Supplementary file 1 [file diagnostics-13-02192-s001.zip › Figure S2. Triton- and SDS-soluble alpha-synuclein in A53T transgenic mice (full blots) (2).tif]
